# Clinical and social determinants of cardiovascular risk in a population with chronic noncommunicable diseases in Maicao, La Guajira, Colombia: 2024

**DOI:** 10.1371/journal.pone.0340101

**Published:** 2025-12-31

**Authors:** Paula Tatiana Angarita-Melo, Karen Panche-Castellanos, Víctor Zein Rizo-Tello, Ana Yibby Forero-Torres, Alexandra Porras-Ramírez

**Affiliations:** 1 Department of Epidemiology, School of Medicine, El Bosque University, Bogotá, Cundinamarca, Colombia; 2 Nutrition Group, Public Health Research Directorate, National Institute of Health, Bogotá, Cundinamarca, Colombia; 3 Community Medicine and Collective Health Research Group, School of Medicine, El Bosque University, Bogotá, Cundinamarca, Colombia; The Chinese University of Hong Kong, HONG KONG

## Abstract

**Aim:**

To determine the independent association and quantify the magnitude of influence of clinical and social determinants on the ten-year cardiovascular disease risk (estimated using the Framingham scale) in individuals diagnosed with arterial hypertension or type 2 diabetes mellitus in Maicao, La Guajira.

**Methods:**

A cross-sectional analytical study was conducted among 273 adults enrolled in noncommunicable disease programs. Anthropometric, biochemical, and sociodemographic data were collected using standardized instruments. Multivariable logistic regression was used to identify and quantify factors associated with high cardiovascular disease risk (defined as 10% or greater Framingham score).

**Results:**

The prevalence of high ten-year cardiovascular disease risk was 16.9%, being significantly higher in women (23.5%) compared to men (3.3%). The multivariable analysis quantified the influence of the determinants. Key clinical factors associated with the highest magnitude of risk were Type 2 Diabetes Mellitus (Adjusted Odds Ratio: 21.87) and High Blood Pressure (Adjusted Odds Ratio: 16.04). The independent effect of a social determinant, receiving a monthly salary, was also strongly associated with high risk (Adjusted Odds Ratio: 4.62). Conversely, being male and having normal High Density Lipoprotein cholesterol levels were identified as protective factors.

**Conclusion:**

This study quantifies that, in addition to the strong influence of traditional clinical factors (T2DM and HBP), social determinants such as income-related work status exert a significant and independent effect on cardiovascular risk in this vulnerable population. The findings underscore the critical need for integrated public health strategies in Maicao, La Guajira, that not only target metabolic control but also effectively address structural social and gender inequalities to achieve a meaningful reduction in the cardiovascular disease burden.

## Introduction

Cardiovascular diseases (CVDs) are the leading cause of death, disability, and disease burden worldwide. Between 1990 and 2019, the number of prevalent CVD cases doubled, from 271 million to 523 million; deaths increased from 12.1 million to 18.6 million, and disability-adjusted life years (DALYs) rose from 143 million to 182 million [1]. Beyond their clinical impact, CVDs generate a substantial economic burden, with estimated healthcare costs exceeding €280 billion in Europe and $400 billion in the United States annually [2,3].

Since the mid-20th century, extensive research has identified major risk factors -such as smoking, hypertension, diabetes, dyslipidemia, and a sedentary behavior responsible for the increasing CVD burden [4]. However, these factors do not act in isolation:, in low- and middle-income countries (LMICs), structural inequities and limited access to preventive and therapeutic health services exacerbate the risk, leading to earlier onset and worse outcomes [5].In Colombia, according to the Vital Statistics Report (EEVV) of the National Administrative Department of Statistics (DANE), CVD remained the leading cause of death between 2023 and 2024, mainly due to ischemic heart disease (17.2%) and cerebrovascular diseases (6.4%) [6]. The economic impact is also considerable:, the average cost of each coronary event for 2014 exceeded 23 million pesos [7]. and the total annual cost associated with CVD reached approximately 2.5 trillion pesos by 2021 [8], positioning it as a major public health priority.

La Guajira, a department in northern Colombia known for its cultural diversity and challenging geographic conditions, faces deep structural inequities. Limited healthcare infrastructure, a shortage of trained health personnel, geographical barriers and poor health information systems restrict access to timely and adequate care [9,10]. This structural vulnerability is reflected in high mortality rates and increased demand for health services. In 2021, circulatory system diseases accounted for 117.25 deaths per 100,000 inhabitants, including ischemic heart disease (57.21), cerebrovascular disease (25.08), and hypertensive disease (19.96). By 2022, cardiovascular diseases represented a significant burden on the health system, with 1,933,779 health care visits, primarily among women (1,244,738). [11]. The municipality of Maicao is particularly affected, recording the highest proportion of care for individuals with chronic non-communicable diseases (NCDs), the highest mortality rate from circulatory system diseases (152.70 per 100,000 inhabitants adjusted by age)and one of the highest mortality rates for diabetes at the departmental level (18.09 per 100 thousand inhabitants adjusted by age) in 2023 [12].

To address these disparities, the Colombian Ten-Year Public Health Plan 2022–2031 and Resolution 429 of 2016 establishes the strategy for the provision of socio-health services with cultural and territorial adaptations for subjects of special protection and people in contexts, situations or conditions of greater vulnerability, with cardio-cerebrovascular-metabolic conditions, including hypertension and type 2 diabetes mellitus) [13]. However, despite the recognized vulnerability of this population, there are no studies in La Guajira that systematically quantify cardiovascular risk or explore how clinical and social determinants interact to exacerbate it.

Various models have been developed to estimate cardiovascular disease risk, among which the Framingham Risk Score stands out as a globally validated tool for estimating the 10-year probability of a major cardiovascular event [14] Crucially, the version adjusted and validated for the Colombian population is the established national reference for risk stratification [15]. Nonetheless, conventional models are primarily focused on clinical variables and may not fully reflect the amplified risk in populations facing structural inequities and health access barriers [16]

Therefore, the objective of this study was to determine the independent association and quantify the magnitude of influence of clinical and social determinants on the ten-year cardiovascular disease risk (estimated using the Framingham scale) in individuals diagnosed with arterial hypertension or type 2 diabetes mellitus in Maicao, La Guajira. To our knowledge, this is the first study in Colombia to provide non-trivial, quantifiable evidence of this synergistic effect in a highly vulnerable border population, offering critical insights to guide culturally and contextually appropriate public health interventions.

## Materials and methods

### Ethics

The study was conducted in accordance with the principles established in the Declaration of Helsinki and Colombian national regulations (Resolution 8430 of 1993). The protocol was approved by the Institutional Ethics Committee of the National Institute of Health (INS) (Code 16–2023, approval record 21). All participants received detailed information about the study objectives and procedures and signed informed written consent prior to their inclusion.

### Study Population

A cross-sectional, analytical study was conducted. The target population consisted of individuals 18 years of age or older with a previous diagnosis of Type 2 Diabetes Mellitus (T2DM) and/or Arterial Hypertension (HTA).These participants were enrolled in the NCD programs of nine Health Care Providers (HPIs)and resided in both urban and rural areas of the municipality. The recruitment process took place between June 2 and May 29, 2024. A non-probability purposive sampling strategy was applied, based on the accessibility and availability of participants through invitations managed by the participating HCPs and community health promoters.

### Inclusion and Exclusion Criteria

Eligible: Individuals who attended scheduled monthly or quarterly check-ups during the previous semester.

Excluded: Participants with physical limitations that would prevent the proper taking anthropometric measurements or venous blood samples

### Data Collection

Data collection was conducted through a modular survey questionnaire in Spanish administered by trained healthcare professional using CSPro software, version 8. The instrument included validated items to assess sociodemographic characteristics, personal and family history, and lifestyle behaviors (e.g., tobacco and alcohol use).

### Measurements

#### Anthropometric and Clinical Measurements.

Anthropometric measurements of weight, height, and waist circumference were performed according to the International Society for the Advancement of Kinanthropometry (ISAK, 2012) [17]. Classification followed World Health Organization (WHO) standards and Colombian Resolution 2465 of 2016 [18,19]. Blood pressure was measured following the European Society of Cardiology (ESC) 2024 Guidelines, with hypertension being defined as a systolic blood pressure (SBP) ≥140 mmHg or a diastolic blood pressure (DBP) ≥90 mmHg [20].

#### Biochemical Measurements and Risk Estimation.

Fasting blood glucose was determined via capillary photometry using HemoCue® Glucose 201. Glycated hemoglobin (HbA1c) was measured with the NYCOCARD® HbA1c kit (Abbott® Laboratories).

The lipid profile (total cholesterol, triglycerides high-density lipoprotein [HDL], and low-density lipoprotein [LDL]) was determined colorimetrically using the DimensionRxL MaX® analyzer (Siemens Healthineers AG). Cutoff points followed the 2024 Colombian Consensus on dyslipidemia (total cholesterol ≥200 mg/dL, triglycerides ≥150 mg/dL, LDL ≥ 130 mg/dL) [21].

Cardiovascular risk was estimated using the Framingham Risk Score, adapted and validated for the Colombian population [15]. This tool estimates the 10-year probability of developing a major cardiovascular event. This method is recommended by Resolution 3280 of 2018 of the Ministry of Health for primary care risk assessment in Colombia [22].

### Statistical analysis

All statistical analyses were conducted using Stata® Statistical Software, version 12.0 (StataCorp LP, College Station, TX, USA). Descriptive and inferential analyses followed the STROBE and SAMPL guidelines for cross-sectional studies to ensure transparency and reproducibility.

#### Data processing and assumption testing.

Prior to analysis, data were examined for completeness, consistency, and normality. Continuous variables were visually inspected using histograms and Q–Q plots to assess distribution, and the Shapiro–Wilk test was applied when appropriate. Variables with non-normal distributions were summarized as medians with interquartile ranges (IQRs) and analyzed using nonparametric tests.

Multicollinearity among predictor variables was evaluated using the Variance Inflation Factor (VIF), with VIF > 10 indicating exclusion from multivariable models. Missing data (<5% for all variables) were handled by listwise deletion, as no systematic pattern was identified.

No mathematical data transformations were necessary; however, several variables were recategorized according to established clinical and epidemiological cutoffs. Specifically, body mass index (BMI), waist circumference and blood pressure were categorized. These categorizations were applied to facilitate interpretation and align with national clinical criteria. For age, a log transformation was applied to improve linearity with the logit function.

#### Descriptive and bivariate analysis.

Categorical variables were summarized as absolute and relative frequencies, and continuous variables as means (± standard deviation) or medians (IQR), depending on distribution. Associations between categorical variables were evaluated using Pearson’s Chi-square test. Comparisons of continuous variables between groups were performed using Student’s two-tailed t-test for independent samples when normally distributed, or the Mann–Whitney U test for nonparametric data. The significance level was defined as α = 0.05 (two-tailed).

#### Multivariable modeling.

To identify independent factors associated with high cardiovascular disease (CVD) risk, was operationalized as a dichotomous variable for the binary logistic regression analysis. Following the interpretation of the Framingham risk Table proposed by Álvarez Cosmea [23], the cutoff point was set at ≥ 10%; therefore, risk < 10% was classified as “no risk” and risk ≥ 10% as “at risk”. The research design was between-subjects, and independent variables were selected a priori based on biological plausibility and previous literature evidence. A forward stepwise selection approach was applied, with entry and removal criteria set at p < 0.05 and p > 0.10, respectively. Adjusted Odds Ratios (aORs) with 95% confidence intervals (CIs) were estimated.

Model specification was evaluated using the Link test, and calibration with the Hosmer–Lemeshow goodness-of-fit test. Discrimination was assessed through the Area Under the Receiver Operating Characteristic (ROC) curve (AUC). Multicollinearity, residual diagnostics, and interaction terms were verified and reported.

### AI statement

During the preparation of this manuscript, the artificial intelligence tool ChatGPT (OpenAI) was used exclusively for editorial purposes, to support writing, improve style, and correct grammar. The authors critically reviewed and validated all generated content, ensuring accuracy, consistency, and correspondence with the study’s findings, interpretations, and conclusions. This tool was not used to generate, modify, or analyze research data.

## Results

### Social, demographic, and economic variables

A total of 273 individuals were included in the study. Social, demographic and economic characteristics are summarized in Table 1, with a predominance of women (67.03%). The mean age was 55.65 years (SD ± 14.54), with no significant sex differences. Nearly half of the participants (46.15%) reported not belonging to any ethnic group, 36.26% self-identified as Indigenous, and a smaller proportion as Afro-Colombian or Raizal. Most participants 83.50% of participants lived in urban areas, and over 40% were single, with significant differences by sex (p < 0.01).

**Table 1:** Social, demographic and economic characteristics of the participants by sex.

	Man	Woman	Total	P-value
	n (%)	n (%)	n (%)	
	90 (32.96)	183 (67.03)	273 (100.00)	
Age (years)*	59.00 ± 12.68	55.65 ± 14.54	56.75 ± 14.02	
** < 40**	5 (5.55)	24 (13.11)	29 (10.62)	0.23
**40-59**	38 (42.22)	79 (43.17)	117 (42.86)	
**60-79**	42 (46.67)	73 (39.89)	115 (42.12)	
** > 80**	5 (5.55)	7 (3.83)	12 (4.39)	
**Ethnicity**				
**Afro-Colombian**	21 (23.33)	25 (13.66)	46 (16.85)	0.16
**Indigenous**	27 (30.00)	72 (39.34)	99 (36.26)	
**Raizal**	1 (1.11)	1 (0.54)	2 (0.73)	
**None**	41 (45.56)	85 (46.45)	126 (46.15)	
**Residence**				
**Rural**	11 (12.22)	34 (18.58)	45 (16.48)	0.18
**Urban**	79 (87.78)	149 (81.42)	228 (83.52)	
**Marital status**				
**Without a partner**	19 (21.11)	94 (51.37)	113 (41.39)	**< 0.01**
**With a partner**	71 (78.89)	89 (48.63)	160 (58.61)	
**Studies**				0.56
**No schooling**	8 (8.88)	17 (9.34)	25 (9.19)	
**Primary education**	31 (34.45)	78 (42.86)	109 (40.07)	
**Secondary education**	31 (34.45)	54 (29.67)	85 (31.25)	
**Higher education**	20 (22.22)	33 (18.13)	53 (19.49)	
**Affiliation to the SGSSS**				0.32
**Contributory**	14 (15.56)	32 (17.49)	46 (16.85)	
**Subsidized**	74 (82.22)	150 (81.97)	224 (82.05)	
**Economic activity**				0.42
**Employed**	72 (80.00)	153 (83.61)	225 (82.42)	
**Unemployed**	14 (15.56)	19 (10.38)	33 (12.09)	
**Other**	4 (4.44)	11 (6.01)	15 (5.50)	
**Income level**				**< 0.05**
** < 1 CLMMW**	41 (45.96)	56 (30.60)	97 (35.53)	
**1 CLMMW**	21 (23.33)	18 (9.83)	39 (14.29)	
** > 1 CLMMW**	6 (6.67)	6 (3.27)	12 (4.39)	
**Receive monthly money**				
**Yes**	41 (45.56)	58 (31.69)	99 (36.26)	**< 0.05**
**No**	49 (54.45)	125 (68.31)	174 (63.74)	

*Mean ± SD

SGSSS: General Health Social Security System of Colombia; CLMMW: Current Legal Monthly Minimum Wage

Regarding economic conditions, only 4.39% reported receiving income greater than one current legal minimum wage (1 CLMMW= USD 323.3). Significant sex differences were also observed in income levels (p < 0.05).

### Clinical status of participants

As exhibited in Table 2 most participants presented general obesity, according to the body mass index (BMI), with a median of 29.22 (IQR 25.56–33.49). Waist circumference was significantly higher in women (p < 0.01) with a greater prevalence of central obesity among them.

**Table 2:** Clinical status of participants by sex.

	Man	Woman	Total	P-value
	n (%)	n (%)	n (%)	
	90 (32.96)	183 (67.03)	273 (100.00)	
**IMC†**	28.37 (25.26–32.98)	30.25 (25.87–33.60)	29.22 (25.56–33.49)	
**Normal**	33 (36.67)	50 (27.32)	83 (30.40)	
**Overweight**	25 (27.78)	48 (26.23)	73 (26.74)	0.27
**Obese**	32 (35.55)	85 (46.45)	117 (42.86)	
Waist circumference*	100.37 ± 14.69	94.62 ± 13.01	96.52 ± 13.83	
**Central obesity**	66 (73.33)	163 (89.07)	229 (83.88)	**< 0.01**
**Normal**	24 (26.67)	20 (10.93)	44 (24.04)	
**Blood pressure**				
**Optimal**	33 (36.67)	110 (60.11)	143 (52.38)	
**Normal**	1 (1.11)	15 (8.19)	16 (5.86)	**< 0.01**
**Normal-high**	30 (33.33)	21 (11.48)	51 (18.68)	
**Grade 1 hypertension**	21 (23.33)	29 (15.85)	50 (18.32)	
**Grade 2 hypertension**	4 (4.44)	6 (3.27)	10 (3.66)	
**Grade 3 hypertension**	1 (1.11)	2 (1.09)	3 (1.09)	
**T2DM report**	27 (30.00)	64 (34.97)	91 (33.33)	0.41
**HbA1c > 7**	8 (29.63)	27 (42.19)	35 (38.46)	0.26
**HDL Cholesterol**				
** > 40 mg/dL**	62 (68.89)	148 (80.87)	210 (76.92)	**< 0.05**
**LDL Cholesterol**				
**Optimal**	49 (54.44)	95 (51.91)	144 (52.75)	
**Near optimal**	21 (23.33)	47 (25.68)	68 (24.91)	0.94
**Borderline high**	12 (13.33)	22 (12.02)	34 (12.45)	
**High**	6 (6.67)	12 (6.55)	18 (6.59)	
**Very high**	2 (2.22)	7 (3.82)	9 (3.29)	

*Mean ± SD

†Median (IQR)

BMI: Body mass index, HbAc1: Glycosylated hemoglobin, LDL: Low-density lipoprotein, T2DM:Type 2 Diabetes Mellitus

A considerable proportion of participants had grade 1 and 2 hypertension, particularly men, who also presented higher mean blood pressure values (p < 0.01). One-third of the population reported a previous diagnosis of diabetes mellitus, with suboptimal glycemic control (HbA1c > 7%) in 38.46% of these cases.

Regarding lipid profile, a higher proportion of women had HDL cholesterol levels in the desired range (p < 0.05); while LDL cholesterol levels did not differ significantly by sex.

### Coronary artery disease risk according to the Framingham score

The categorization of 10-year cardiovascular risk using the Framingham score revealed that 16.85% of the total study population presented an elevated risk. The prevalence of this risk was significantly higher in women, reaching 23.5% compared to 3.33% observed in men (p < 0.01). Bivariate analysis presented in Table 3 showed that this high-risk prevalence was significantly associated with adiposity measurements. Specifically, central obesity was a strongly associated factor (20.09% high risk vs. 0.00% in normal waist circumference) (p < 0.01), as was general obesity by BMI (23.62% in the Obesity category) (p < 0.05). It is important to note that no cases of high cardiovascular risk were identified among participants aged 80 years or older. Other clinical variables also showed patterns of association: the risk was considerably higher in people with a diabetes diagnosis (34.07% high risk) and in those with Grade 2 (40.00%) and Grade 3 (33.33%) hypertension.

**Table 3:** Categorization of cardiovascular risk in study participants.

	Cardiovascular risk		P-value
	Yes	No	
	n (%)	n (%)	
	46 (16.85%)	227(83.15%)	
**Sex**			
**Man**	3 (3.33)	87 (96.67)	< 0.01
**woman**	43 (23.5)	140 (76.50)	
**Age**			
** < 40**	7 (24.14)	22 (75.86)	0.07
**40-59**	25 (21.37)	92 (78.63)	
**60-79**	14 (12.17)	101 (87.83)	
** > 80**	0 (0.00)	12 (100.00)	
**Marital status**			
**Without a partner**	24 (21.24)	89 (78.76)	0.10
**With a partner**	22 (13.75)	138 (86.25)	
**BMI**			
**Normal**	6 (11.32)	47 (88.68)	< 0.05
**Overweight**	10 (10.99)	81 (89.01)	
**Obese**	30 (23.62)	97 (76.38)	
**Waist circumference**			
**Normal**	0 (0.00)	44 (100.00)	< 0.01
**Central obesity**	46 (20.09)	183 (79.91)	
**Blood pressure**			
**Optimal**	17 (11.89)	126 (88.11)	0.07
**Normal**	3 (18.75)	13 (81.25)	
**High-Normal**	8 (15.69)	43 (84.31)	
**Grade 1 Hypertension**	13 (26.00)	37 (74.00)	
**Grade 2 Hypertension**	4 (40.00)	6 (60.00)	
**Grade 3 Hypertension**	1 (33.33)	2 (66.67)	
**T2DM**			
**T2DM Report**	31 (34.07)	60 (65.93)	0.06
**HbA1c > 7**	16 (45.71)	19 (54.29)	
**Cholesterol HDL**			
** > 40 mg/dL**	31 (14.76)	179 (85.24)	0.09

BMI: body mass index, HbA1c: Glycosylated hemoglobin, HDL: High-density lipoprotein;T2DM:Type 2 Diabetes Mellitus

### Multivariate analysis of clinical, biological, and social determinants of cardiovascular risk

In the multivariate logistic regression model presented in the Table 4 the factors most strongly associated with an increased likelihood of cardiovascular disease (CVD) risk were type 2 diabetes mellitus (OR: 21.87; 95% CI: 6.96–68.71; p < 0.001), high blood pressure (OR: 16.04; 95% CI: 3.15–81.69; p = 0.001), and receiving a monthly salary (OR = 4.62; 95% CI: 1.56–13.64; p = 0.006). Conversely, normal HDL cholesterol levels (OR: 0.21; 95% CI: 0.07–0.61; p = 0.004), male sex (OR: 0.03; 95% CI: 0.007–0.16; p < 0.001), and older age (OR: 0.92; 95% CI: 0.88–0.96; p < 0.001) acted as protective factors.

**Table 4:** Multivariate logistic regression model of factors associated with cardiovascular disease (CVD) risk.

Variable	OR	Standard error	z	P-Value	IC 95%	
**Sex**						
**Male**	**0.033**	**0.027**	**−4.20**	**0.001**	**0.007**	**0.162**
**HBP**	**16.039**	**13.321**	**3.34**	**0.001**	**3.149**	**81.689**
**Type 2 Diabetes**	**21.865**	**12.774**	**5.28**	**0.001**	**6.958**	**68.711**
**Occupation**						
** Unemployed**	2.635	1.996	1.28	0.201	0.597	11.630
** Other**	1.081	1.357	0.06	0.951	0.092	12.676
**Receives monthly salary**	**4.618**	**2.551**	**2.77**	**0.006**	**1.564**	**13.636**
**Educational level**						
** Elementary education**	0.335	0.269	−1.36	0.174	0.069	1.621
** Secondary education**	0.392	0.330	−1.11	0.266	0.075	2.041
** Higher education**	0.211	0.202	−1.63	0.104	0.032	1.375
** Marital status (with a partner)**	1.296	0.681	0.49	0.621	0.463	3.631
** Area of residence (urban)**	2.892	2.001	1.54	0.125	0.745	11.224
**Ethnicity**						
** Indigenous**	1.514	1.197	0.52	0.600	0.322	7.126
** Other**	0.914	0.702	−0.12	0.906	0.202	4.123
** Raizal**	34.051	204.318	0.59	0.557	0.0003	4,361,171
** HDL cholesterol (normal)**	**0.207**	**0.113**	**−2.87**	**0.004**	**0.071**	**0.606**
**LDL cholesterol**						
** High**	0.686	0.975	−0.27	0.791	0.042	11.121
** Borderline high**	0.832	1.106	−0.14	0.890	0.061	11.275
** Near optimal**	2.955	3.691	0.87	0.386	0.256	34.178
** Optimal**	0.453	0.563	−0.64	0.524	0.040	5.173
** Age (log-transformed)**	**0.923**	**0.020**	**−3.67**	**0.001**	**0.885**	**0.963**
** Constant (_cons)**	6.800	14.297	0.91	0.362	0.110	418.893

Other:Afro-Colombian, None.

The overall model fit was satisfactory (LR χ² (20) = 108.30, p < 0.001), explaining an acceptable proportion of the outcome variability (McFadden pseudo R² = 0.438). Model specification and calibration were appropriate (Link test: p = 0.086; Hosmer–Lemeshow: p = 1.000).

**Fig 1:**
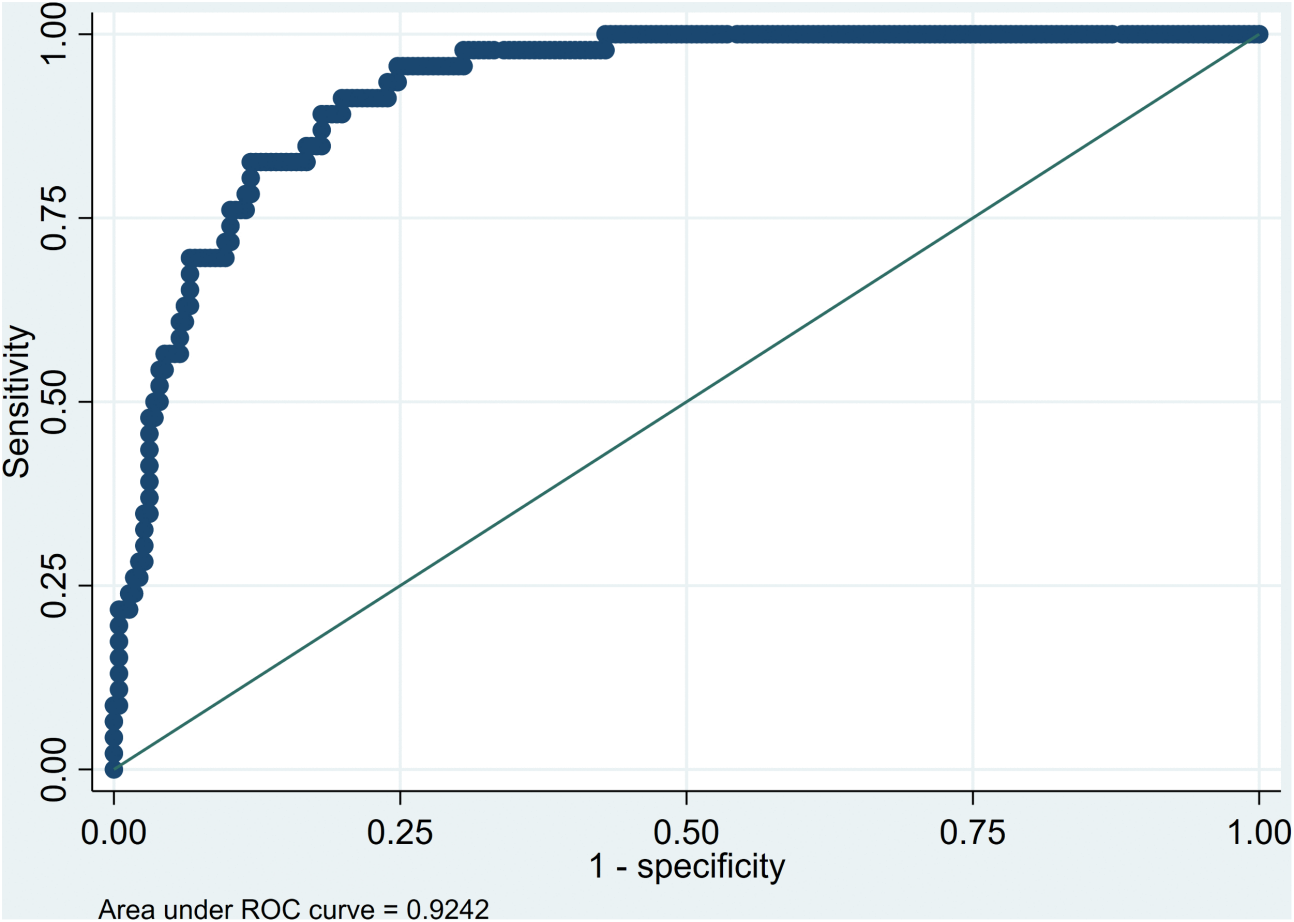
Receiver Operating Characteristic (ROC) curve of the multivariate logistic regression model for cardiovascular disease (CVD) risk.

The explanatory logistic regression model demonstrated an excellent ability to distinguish between individuals with and without cardiovascular risk. The area under the receiver operating characteristic (ROC) curve was 0.926 (Fig 1), indicating high discriminatory capacity according to conventional classification criteria. The classification matrix showed a sensitivity of 54.35%, a specificity of 95.58%, and an overall correct classification rate of 88.6%, confirming the model’s robustness and internal consistency. No multicollinearity issues were identified among predictors (mean VIF: 2.54). These findings confirm that the model adequately captured the combined effects of clinical and social determinants on ten-year cardiovascular disease risk in individuals with hypertension or type 2 diabetes mellitus in Maicao, La Guajira.

## Discussion

The present study identified statistically significant associations between the development of cardiovascular disease (CVD) and various risk factors, including female sex, obesity, and high blood pressure.

Regarding obesity, gender differences were observed. A higher proportion of women were classified as obese based on a BMI ≥ 30 (46.45% vs. 35.55%) and waist circumference ≥ 80 (89.07% vs. 73.33%) compared to men, although without statistically significant differences. Despite this, it is important to note that women are primarily affected by obesity. Beyond the biological factor associated with greater adiposity, it may be due to socioeconomic, cultural, or other differential factors that primarily affect women. As has been found in previous studies, obesity has been associated with low educational attainment, unfavorable socioeconomic conditions, and low income, especially in women [24]. Non-traditional risk factors specific to women include excessive pregnancy weight gain, preeclampsia, gestational diabetes, premature birth, and menopause. The highest prevalence of obesity in women is primarily evident in adulthood, peaking between the ages of 60 and 64. These sex-dependent differences in the distribution of obesity have been attributed to multiple factors, including differences in dietary patterns, energy expenditure, health-related behaviors, and gender-specific sociocultural and environmental conditions [25].

Chen et al. examined a cohort of 2,683 postmenopausal women with a body mass index (BMI) within the normal range and found that greater accumulation of fat at the trunk level, accompanied by a lower proportion of fat in the lower extremities, was significantly associated with an increased risk of CVD, and that the total fat mass-to-body weight ratio was not significantly associated with cardiovascular disease (CVD). Given the above, our study may have included women with a different cardiovascular risk than could be measured solely through body mass index, so studies that take into account the distribution of adipose tissue are suggested [26].

In line with this, it has been reported that the burden of obesity and metabolic risk factors in women is higher in rural areas and vulnerable contexts in Latin America. An analysis of the PURE Colombia study cohort showed that women had a higher prevalence of abdominal obesity and lower physical activity than men, which contributes to early cardiovascular risk in these populations [27]. Furthermore, the Pan American Health Organization (PAHO) and other studies highlight that, in contexts of poverty, women face structural barriers to accessing healthy diets and physical activity, which perpetuates the obesity-poverty-disease cycle [28,29]. However, a higher proportion of women with normal HDL cholesterol levels was observed compared to men (80.87% vs. 68.89%; p < 0.05). Despite the high prevalence of obesity in the female population, it is important to highlight that the median age of women was between 25.87 and 33.60 years. According to the literature, estradiol (E2) promotes an increase in HDL cholesterol concentration in women, who present elevated E2 levels in their adult years prior to menopause [30]. Considering the age range of the women, this could be a physiological explanation for the observed levels; however, other types of studies are needed, including sociodemographic and cultural aspects that may also influence the lipid profile of the participants.

Another risk factor for CVD was hypertension, with a higher proportion of individuals with grade 1 (26.00%), 2 (40.00%), and 3 (33.33%) hypertension (p = 0.07), and a higher prevalence in men. According to Zurique-Sánchez et al., the prevalence of HBP is slightly higher in men than in women (29% [95% CI 23–37%; p < 0.001] vs 28% [95% CI: 21–36%; p < 0.001), although the results of this analysis had a high degree of heterogeneity [28], which implies that the differences between studies may be due to conditions specific to each population, such as diet. In La Guajira, other research reported that the main dietary patterns are the so-called “industrial” (sweets, fast food, soft drinks) and “grill/drink” (alcoholic beverages, grilled foods, energy drinks). Multivariate analysis identified that individuals with HTN are more likely to present industrial (OR 1.202 [95% CI: 1.074–1.346; p = 0.001]) or grilled/drink (OR 1.247 [95% CI: 1.149–1.354; p < 0.001]) dietary patterns [31].

From a cultural perspective, the typical diet of this community frequently includes fish fried in recycled corn oil or lard, in addition to other traditional foods. This type of preparation could influence the population’s lipid composition, depending on both the predominant type of fat and its interaction with other metabolic factors. This could explain the differences in the protective effect of HDL cholesterol observed in this study and, in part, the identified cardiovascular risk. This aligns with previous studies reporting that fish is a significant dietary source of omega-3 long-chain polyunsaturated fatty acids (n-3 PUFAs), and its regular consumption is associated with a reduced risk of developing cardiovascular diseases such as acute myocardial infarction, hypertension, atherosclerosis, and stroke. This is due to its anti-inflammatory properties and its ability to reduce plasma triglyceride levels, as well as its potential vasodilatory, antiarrhythmic, and antihypertensive effects [32]. Similarly, a meta-analysis showed that higher fish consumption is significantly associated with a lower incidence of ischemic heart disease (relative risk [RR]: 0.91; 95% confidence interval [95% CI]: 0.84–0.97; I² = 47.4%). Likewise, the study reported that higher fish intake is significantly associated with a reduction in mortality from ischemic heart disease (RR: 0.85; 95% CI: 0.77–0.94; I² = 51.3%) [33].

Contrary to what was reported in the systematic review published by Aljuraiban GS et al., which showed that the adoption of the DASH dietary pattern, supported by high-quality randomized controlled trials (RCTs), produces a significant reduction in both systolic blood pressure (SBP) and diastolic blood pressure (DBP). This effect was also observed with several of its components, such as high consumption of fruits, vegetables, whole grains, legumes, nuts, seeds, lean red meat, poultry, and products rich in lactotripeptides. Likewise, an association was identified between increased urinary potassium excretion and lower sodium intake with lower blood pressure values. Notably, the magnitude of the DASH pattern effect was comparable to that obtained with antihypertensive drug treatment, with similar results observed in the case of the Mediterranean diet. The inhabitants of La Guajira have a way of eating adapted to their culture and the region’s unique agriculture, which may not be the healthiest and can lead to overweight and obesity, high blood pressure, and increased cardiovascular risk [34].

Men in the present study had a lower risk of CVD (OR 0.033) compared to women. The literature reports that men tend to have a higher risk of CVD compared to women until menopause, at which point women’s risk increases, even exceeding that of men [30]. However, it is important to highlight that this could be related to certain factors within the population, such as socioeconomic inequalities, which may be reflected in an unhealthy diet that favors the onset of CVD [35].

The results for high blood pressure and T2DM were consistent with what has been previously established in the literature as risk factors for CVD. In contrast, the variable of monthly income was not consistent with what has been previously described in the literature, which generally associates low income with a higher risk of CVD [36,37]. In Colombia, there are reports associating socioeconomic inequality with the risk of high blood pressure [38,39]. This could be because people with higher monthly incomes might adopt certain culturally accepted consumption habits, such as smoking or drinking alcohol, or even modify their diet to reflect negative dietary patterns [40,41]. Therefore, these factors would have a greater impact on this population specifically, as their basic conditions are not covered, and they lack the necessary resources for timely diagnosis, treatment, and adequate follow-up, which would lead to a greater disease burden and higher mortality.

The factors found in this study to have a protective effect were a normal HDL level (OR 0.21; 95% CI: 0.07–0.61; p = 0.004). Authors such as Hovingh et al. report that the concept of HDL and its value as a predictor of coronary risk is unshakeable, with various studies demonstrating an inverse association between HDL levels and CVD risk [42]. And notably, age was inversely associated with cardiovascular risk, which contrasts with conventional evidence that points to age as a non-modifiable and cumulative risk factor for cardiovascular disease. In this population, each additional year of age was associated with a slight decrease in estimated cardiovascular risk (OR = 0.92; 95% CI: 0.88–0.96; p < 0.001).

This apparent paradox could be interpreted from several perspectives. On the one hand, older adults who remain free of cardiovascular events until advanced stages could represent a resilient cohort, with healthier lifestyles or lower cumulative exposure to contemporary risk factors, such as high consumption of ultra-processed foods or a sedentary lifestyle, which more intensely affect young adults. Several studies show that high BMI trajectories from childhood are associated with an unfavorable cardiometabolic profile in early adulthood, even in the absence of overt childhood obesity [40,43]. However, it could also correspond to a “survival selection” bias, since, in high-risk settings such as the aforementioned municipality of Maicao, those adults who survive could present a healthier health profile or even be underdiagnosed due to limited access to diagnostic tests and clinical monitoring [44].

However, it is possible that the younger age group in the present study is experiencing a disproportionate burden of cardiovascular risk due to structural determinants such as the food environment, urbanization, and social inequalities, which have driven an increase in cardiovascular risk in young people, which could increase their exposure to risk factors in early life [45]. This pattern is particularly relevant in contexts of social vulnerability, where the nutritional transition has driven widespread consumption of ultra-processed foods [40].

The latest update of the Colombian Nutritional Profile Study (COPEN) reported that among urban adults, lower socioeconomic status in women was associated with higher caloric intake from sugary beverages (10.6% vs. 6.6% of the total) [46], reflecting the prevalence of unhealthy dietary habits even in later stages of young adulthood. The region has undergone a marked epidemiological shift, moving from malnutrition to overweight and obesity in just a few decades. A systematic review published in 2025 reported a prevalence of abdominal obesity of approximately 60%, with significant differences between men and women and a clear link to highly obesogenic environments in Latin America [47].

In the present study, we sought to evaluate ethnicity as a possible risk factor for the development of CVD. However, no associations were observed. This is consistent with studies such as that of López-López et al., who reported that the prevalence of hypertension in ethnic communities, such as Afro-descendant communities, is more closely related to socioeconomic factors, such as low educational levels, than to ethnicity per se [27]. Likewise, other authors have reported that the risk of CVD in individuals from this community is completely attenuated after adjusting for variables such as economic deprivation. Therefore, ethnic differences in CVD risk are likely due to inequalities rather than biological factors [48]. In the Indigenous population, on the other hand, there are no reports on HTN and socioeconomic factors. Therefore, it is imperative that studies be conducted to establish the possible association between socioeconomic conditions and inequalities in the Indigenous population affected by HTN.

Finally, it is important to highlight that this study used the Framingham Risk Score, which, although widely used, has limitations such as underestimating risk in young people and originating in the Caucasian American population, which impedes its validity in other groups with racial, social, or economic differences [49]. In turn, this tool does not consider structural factors that influence the increase in risk in population groups, especially those with greater vulnerability. Recent literature suggests that cardiovascular risk prediction models, such as SCORE2, developed in European contexts, could also underestimate the real risk in vulnerable populations in Latin America, where social determinants and structural inequalities play a more accentuated role in the genesis of these diseases [50]. Therefore, the need for contextualized prediction instruments and prevention strategies, validated in specific contexts that take into account early exposure and the accumulated social vulnerability of the study population is emphasized [29].In conclusion, the results of this analysis show that high blood pressure and type 2 diabetes mellitus are the main predictors of cardiovascular risk, presenting strong and statistically significant associations. Male sex showed an inverse association, suggesting greater cardiovascular susceptibility among women in the study population. Among sociodemographic factors, higher education acts as a protective factor against the development of cardiovascular disease, possibly associated with better health habits and access to preventive services.

On the other hand, variables such as occupation, marital status, ethnicity, and area of residence did not show statistically significant associations; however, their indirect influence cannot be ruled out, given their potential impact as social determinants of health.

Overall, the findings highlight the need to implement comprehensive cardiovascular prevention strategies that simultaneously address traditional metabolic factors and social and gender inequalities. Furthermore, it is recommended to continue research in the Maicao (La Guajira) population, considering its sociocultural and demographic characteristics, in order to identify and modify cardiovascular risk factors in a timely manner through interventions supported by public health policies adapted to the local context.

### Limitations

This is an analytical cross-sectional study; therefore, causality cannot be established. Furthermore, there is a selection bias in the inclusion of study participants, as they belong to a follow-up program, which may be serving an unrepresentative fraction of the study population. Similarly, the difficulty certain sectors of the population face in accessing health services may influence the selection of participants. Non-probability sampling prevents inferences from being made about the population. Finally, the use of the Framingham Scale, which is not adapted to the context of the population, may overestimate or underestimate certain factors, as well as variables that are part of the social determinants of health in the study population.

## Conclusions

The significant association of factors with CVD risk in the population was confirmed, such as hypertension and type 2 diabetes. However, these factors may have a much greater impact in this population due to their vulnerable conditions, with a possible amplified effect due to limitations in timely access to diagnosis and treatment. Furthermore, variables such as income, far from being protective, were associated with increased risk in this population, contrary to reports in other populations, suggesting an environmental influence on culturally accepted and potentially harmful consumption patterns. The risk profile showed differences by sex, especially in women, who showed higher prevalence of obesity, lower educational levels, and sustained exposure to structural risk factors.

These results reinforce the need for differentiated approaches to addressing cardiovascular risk, considering the sociocultural and economic context of the population.
